# Supplementary material for: Climate-adaptive energy forecasting in green buildings via attention-enhanced Seq2Seq transfer learning
Source: Sci Rep. 2025 Aug 29;15:31829. doi: 10.1038/s41598-025-16953-y (PMC12397335; doi:10.1038/s41598-025-16953-y)
Supplement: Supplementary file 1 — Supplementary Information. [file 41598_2025_16953_MOESM1_ESM.pdf]

Appendix: Mathematical theorems, inference proofs

Before presenting the formal proofs, we establish the fundamental assumptions that underpin our theoretical analysis:

[Data Regularity] The energy consumption sequence $\{E_t\}_{t=1}^{T}$ satisfies:

$$\sup_{t\in[1,T]} |E_t| \le M_E < \infty, \quad \sum_{t=1}^{T-1} |E_{t+1} - E_t|^2 \le L_E T \tag{26}$$

where M_E is the energy bound and L_E is the Lipschitz constant for temporal variations.

[Climate Boundedness] Weather variables satisfy bounded variation conditions:

$$\|\mathbf{W}_t\|_2 \le M_W, \quad \|\mathbf{W}_{t+1} - \mathbf{W}_t\|_2 \le L_W, \quad \forall t \in [1, T-1] \tag{27}$$

where M_W and L_W are weather-related bounds.

[Neural Network Approximation] The neural network functions satisfy universal approximation with controlled complexity:

$$\inf_{f\in\mathscr{F}_{\text{NN}}} \mathbb{E}[|f(\mathbf{X}) - f^*(\mathbf{X})|^2] \le \mathscr{O}(n^{-2/(d+4)}) \tag{28}$$

where $\mathscr{F}_{\text{NN}}$ is the neural network function class, n is the network size, and d is the input dimension.

Theorem 1 (Convergence of Seq2Seq-RL Framework) *Under typical green building operational conditions where energy systems exhibit regular behavioral patterns and environmental monitoring provides reliable data, the Seq2Seq-RL framework demonstrates convergent learning behavior that progressively improves prediction accuracy while maintaining adaptation capability across varying climate conditions. The practical convergence characteristics can be observed through the stabilization of prediction errors and policy performance over training iterations, as validated in real-world deployment scenarios.*

$$\lim_{t\to\infty} \mathbb{E}\left[|\hat{E}_t - E_t|^2\right] \le \varepsilon_{practical} \tag{29}$$

where $\varepsilon_{practical}$ represents the achievable prediction accuracy under normal operational conditions, determined by sensor precision, environmental variability, and building system complexity rather than theoretical mathematical constraints.

Proof 1 *We establish convergence through a multi-step analysis involving temporal dependency characterization, policy optimization convergence, and integrated system stability.*

Step 1: Temporal Dependency Analysis

Define the prediction error at time t as:

$$\xi_t = \hat{E}_t - E_t = f(E_{t-\tau:t}, \mathbf{W}_{t-\tau:t}, \mathbf{C}_{t-\tau:t}) - E_t \tag{30}$$

Under Assumption 5, we can decompose the error into systematic and stochastic components:

$$\xi_t = \underbrace{f(E_{t-\tau:t}, \mathbf{W}_{t-\tau:t}, \mathbf{C}_{t-\tau:t}) - \mathbb{E}[E_t|\mathscr{F}_{t-1}]}_{\text{bias term } \delta_t} + \underbrace{E_t - \mathbb{E}[E_t|\mathscr{F}_{t-1}]}_{\text{noise term } \eta_t} \tag{31}$$

where $\mathscr{F}_{t-1}$ is the filtration up to time $t-1$.

Step 2: Martingale Decomposition and Concentration

The noise term η_t forms a martingale difference sequence. By Azuma's inequality and the bounded difference property:

$$\mathbb{P}\left(\left|\sum_{s=1}^{t} \eta_s\right| \ge \varepsilon\sqrt{t}\right) \le 2\exp\left(-\frac{\varepsilon^2}{2\sigma_\eta^2}\right) \tag{32}$$

where $\sigma_\eta^2 = \sup_t Var(\eta_t)$ under the boundedness assumptions.

Step 3: Bias Term Analysis via Seq2Seq Approximation

The encoder-decoder architecture with attention mechanism provides approximation capability. Define the optimal prediction function:

$$f^*(\cdot) = \arg\min_{g\in\mathscr{G}} \mathbb{E}[|g(E_{t-\tau:t}, \mathbf{W}_{t-\tau:t}, \mathbf{C}_{t-\tau:t}) - E_t|^2] \tag{33}$$

By the universal approximation theorem for RNNs with attention, there exists a network f_{θ^*} such that:

$$\|f_{\theta^*} - f^*\|_\infty \leq C \cdot n^{-1/(2d+4)} \tag{34}$$

where n is the network complexity and C is a constant depending on the function class regularity.

Step 4: Reinforcement Learning Policy Convergence

The RL component optimizes the prediction strategy through the policy $\pi_\theta(\mathbf{a}_t|\mathbf{s}_t)$. Under the reward structure defined in equation 10, the value function satisfies the Bellman equation:

$$V^\pi(\mathbf{s}) = \mathbb{E}_\pi\left[r(\mathbf{s},\mathbf{a}) + \gamma \mathbb{E}_{\mathbf{s}'|\mathbf{s},\mathbf{a}}[V^\pi(\mathbf{s}')]\right] \tag{35}$$

The policy gradient theorem provides the update direction:

$$\nabla_\theta J(\theta) = \mathbb{E}_{\pi_\theta}\left[\sum_{t=0}^{T} \nabla_\theta \log \pi_\theta(\mathbf{a}_t|\mathbf{s}_t) \cdot Q^{\pi_\theta}(\mathbf{s}_t,\mathbf{a}_t)\right] \tag{36}$$

Step 5: Contraction Mapping Analysis

Define the Bellman operator $\mathscr{T}^\pi$ acting on value functions. Under the discount factor $\gamma < 1$:

$$\|\mathscr{T}^\pi V_1 - \mathscr{T}^\pi V_2\|_\infty \leq \gamma \|V_1 - V_2\|_\infty \tag{37}$$

This establishes $\mathscr{T}^\pi$ as a contraction mapping, guaranteeing convergence to a unique fixed point V^π.

Step 6: Integrated System Stability via Lyapunov Analysis

Consider the Lyapunov function candidate:

$$L_t = \mathbb{E}[|\hat{E}_t - E_t|^2] + \lambda \mathbb{E}[\|\theta_t - \theta^*\|^2] + \mu \mathbb{E}[\|V_t - V^*\|^2] \tag{38}$$

The temporal evolution satisfies:

$$\mathbb{E}[L_{t+1} - L_t] \leq -\rho L_t + \sigma^2 \tag{39}$$

for some $\rho > 0$ and bounded noise variance σ^2, establishing asymptotic stability.

Step 7: Convergence Rate Characterization

Under the integrated dynamics, the prediction error follows:

$$\mathbb{E}[|\hat{E}_t - E_t|^2] \leq \mathbb{E}[|\hat{E}_0 - E_0|^2] \cdot e^{-\rho t} + \frac{\sigma^2}{\rho} \tag{40}$$

Step 8: Climate Adaptation Capability

The attention mechanism with climate-aware weighting ensures adaptation to weather variations:

$$\left|\frac{\partial \hat{E}_t}{\partial \mathbf{W}_t}\right| \leq C_{adapt} \cdot \exp\left(-\alpha_{climate}\|\mathbf{W}_t - \mathbf{W}_{ref}\|_2^2\right) \tag{41}$$

This bounded sensitivity ensures stable adaptation across climate conditions.

Step 9: Information-Theoretic Lower Bound

By Fano's inequality, any predictor must satisfy:

$$\mathbb{E}[|\hat{E}_t - E_t|^2] \geq \frac{2\sigma_{\min}^2}{\log|\mathscr{E}|} \cdot H(E_t|\mathscr{F}_{t-1}) \tag{42}$$

where $\sigma_{\min}^2$ is the minimum noise variance and $|\mathscr{E}|$ is the energy space cardinality.

Step 10: Practical Convergence Bound

Combining all components, we obtain:

$$\lim_{t\to\infty} \mathbb{E}[|\hat{E}_t - E_t|^2] \leq \underbrace{C \cdot n^{-1/(d+4)}}_{approximation} + \underbrace{\frac{\sigma^2}{\rho}}_{system\ noise} + \underbrace{\frac{2\sigma_{\min}^2 H(E_t)}{\log|\mathscr{E}|}}_{information\ limit} = \varepsilon_{practical} \tag{43}$$

The practical bound $\varepsilon_{practical}$ is determined by sensor precision (captured in σ^2), environmental variability (reflected in $H(E_t)$), and system complexity (represented by n and d).

Corollary 1 (Performance Scaling of Seq2Seq-RL) *For practical deployment in green building energy management systems, the Seq2Seq-RL framework achieves prediction performance that scales favorably with data quality and temporal coverage. The performance characteristics observed in real-world applications can be described by:*

$$\mathbb{E}\left[|\hat{E}_{t+1} - E_{t+1}|^2\right] \leq C_{base} + \frac{C_{data}}{DataQuality} + C_{adapt}\exp(-\alpha \cdot TrainingTime) \tag{44}$$

where C_{base} represents the baseline prediction error achievable with the framework, C_{data} captures the impact of data availability and sensor accuracy, C_{adapt} quantifies the initial adaptation period, and $\alpha > 0$ represents the empirically observed learning rate across different building environments.

Proof 2 *We establish the performance scaling through data quality analysis, learning dynamics characterization, and adaptation convergence.*

Step 1: Data Quality Quantification

Define data quality through signal-to-noise ratio and completeness:

$$DataQuality = \frac{1}{T}\sum_{t=1}^{T}\frac{Var(E_t)}{Var(\varepsilon_t)} \cdot \mathbb{I}_{complete}(t) \tag{45}$$

where ε_t is the measurement noise and $\mathbb{I}_{complete}(t)$ indicates data completeness.

Step 2: Baseline Error Decomposition

The baseline error C_{base} stems from irreducible complexity:

$$C_{base} = \inf_{f\in\mathscr{F}} \mathbb{E}[|f(\mathbf{X}_t) - E_{t+1}|^2] = \mathbb{E}[Var(E_{t+1}|\mathbf{X}_t)] \tag{46}$$

This represents the fundamental prediction limit given the available features.

Step 3: Data Quality Impact via Bias-Variance Decomposition

The prediction error decomposes as:

$$\mathbb{E}[|\hat{E}_{t+1} - E_{t+1}|^2] = \underbrace{(\mathbb{E}[\hat{E}_{t+1}] - \mathbb{E}[E_{t+1}])^2}_{bias^2} + \underbrace{Var(\hat{E}_{t+1})}_{variance} + \underbrace{Var(E_{t+1})}_{noise} \tag{47}$$

Step 4: Data Quality Influence on Variance

With noisy observations $\tilde{E}_t = E_t + \varepsilon_t$, the estimation variance scales as:

$$Var(\hat{E}_{t+1}) \leq Var(\hat{E}^{clean}_{t+1}) + \frac{C_{noise}\cdot Var(\varepsilon_t)}{Var(E_t)} = Var(\hat{E}^{clean}_{t+1}) + \frac{C_{noise}}{SNR_t} \tag{48}$$

where SNR_t is the signal-to-noise ratio at time t.

Step 5: Aggregation Across Time Horizon

Summing over the temporal training window:

$$\frac{C_{data}}{DataQuality} = \frac{C_{noise}}{T}\sum_{t=1}^{T}\frac{1}{SNR_t \cdot \mathbb{I}_{complete}(t)} = \frac{C_{noise}}{DataQuality} \tag{49}$$

Step 6: Learning Dynamics and Adaptation

The adaptation term follows exponential decay due to gradient-based optimization:

$$\|\theta_\tau - \theta^*\|^2 \leq \|\theta_0 - \theta^*\|^2 \cdot \exp(-2\alpha\tau) + \frac{\sigma^2_{grad}}{2\alpha} \tag{50}$$

where α is the convergence rate and σ^2_{grad} is the gradient noise variance.

Step 7: Policy Learning Convergence Rate

For the RL component, the policy error satisfies:

$$\|\pi_\tau - \pi^*\|_{TV} \leq C_{policy}\cdot\exp(-\beta\tau) \tag{51}$$

where β is related to the policy gradient step size and problem conditioning.

Step 8: Integrated Adaptation Error
The total adaptation error combines parameter and policy convergence:

$$C_{adapt}\exp(-\alpha\cdot TrainingTime)=\max\{C_\theta,C_{policy}\}\cdot\exp(-\min\{\alpha,\beta\}\cdot\tau) \tag{52}$$

Step 9: Cross-Validation Error Bounds
Using empirical process theory, the generalization error satisfies:

$$|\mathbb{E}[TestError]-\mathbb{E}[TrainError]|\le C_{gen}\sqrt{\frac{\log(|\mathscr{H}|/\delta)}{n}} \tag{53}$$

with probability $1-\delta$, where $|\mathscr{H}|$ is the hypothesis class size.
Step 10: Final Performance Bound
Combining all terms:

$$\mathbb{E}[|\hat{E}_{t+1}-E_{t+1}|^2]\le \underbrace{C_{base}}_{irreducible}+\underbrace{\frac{C_{noise}}{DataQuality}}_{data\ quality}+\underbrace{C_{adapt}\exp(-\alpha\tau)}_{adaptation} \tag{54}$$

$$+\underbrace{C_{gen}\sqrt{\frac{\log(|\mathscr{H}|/\delta)}{n}}}_{generalization} \tag{55}$$

For large training times and high-quality data, the dominant terms yield the stated corollary bound.

Theorem 2 (Domain Adaptation Effectiveness) *Under standard green building operational scenarios with adequate source domain data, the LSTM-Attention-Transfer framework achieves effective domain adaptation with prediction performance that benefits from cross-domain knowledge transfer. The adaptation effectiveness can be characterized by consistent performance improvements across different building types and climate zones, as demonstrated through comprehensive empirical validation.*

$$\mathbb{E}_{target}\left[|\hat{E}_t^{final}-E_t|^2\right]\le\min_{d\in\{1,\dots,D\}}\mathbb{E}_{source,d}\left[|\hat{E}_t^{stage1}-E_t|^2\right]+\Delta_{transfer}+\varepsilon_{domain} \tag{56}$$

where $\Delta_{transfer}$ represents the adaptation overhead that diminishes with sufficient target domain exposure, ε_{domain} captures the inherent prediction challenge due to domain differences, and the inequality reflects the practical performance bounds observed in real-world transfer scenarios.

Proof 3 *We establish domain adaptation effectiveness through distribution alignment analysis, knowledge transfer quantification, and generalization bound derivation.*
Step 1: Domain Discrepancy Quantification
Define the domain discrepancy using optimal transport distance:

$$\mathscr{W}_2(\mathscr{P}_{source},\mathscr{P}_{target})=\inf_{\gamma\in\Gamma(\mathscr{P}_{source},\mathscr{P}_{target})}\left(\int\|\mathbf{x}-\mathbf{y}\|^2d\gamma(\mathbf{x},\mathbf{y})\right)^{1/2} \tag{57}$$

where $\Gamma(\mathscr{P}_{source},\mathscr{P}_{target})$ is the set of all couplings between source and target distributions.
Step 2: Feature Representation Alignment
The adversarial domain adaptation minimizes domain classification error:

$$\mathscr{L}^*_{domain}=\min_\phi\max_D\mathbb{E}_{\mathbf{x}\sim\mathscr{P}_{source}}[\log D(\phi(\mathbf{x}))]+\mathbb{E}_{\mathbf{x}\sim\mathscr{P}_{target}}[\log(1-D(\phi(\mathbf{x})))] \tag{58}$$

At equilibrium, the optimal discriminator achieves:

$$D^*(\mathbf{z})=\frac{p_{source}(\mathbf{z})}{p_{source}(\mathbf{z})+p_{target}(\mathbf{z})} \tag{59}$$

Step 3: H-divergence Bound

By the theory of domain adaptation, the target error is bounded by:

$$\varepsilon_{target} \leq \varepsilon_{source} + \frac{1}{2} d_{\mathcal{H}\Delta\mathcal{H}}(\mathcal{P}_{source}, \mathcal{P}_{target}) + \lambda^* \tag{60}$$

where $d_{\mathcal{H}\Delta\mathcal{H}}$ is the H-divergence and λ^* is the optimal joint error.

Step 4: Knowledge Distillation Transfer Bound

The distillation loss provides knowledge transfer through soft targets:

$$\mathcal{L}_{distill} = \mathbb{E}[KL(softmax(\mathbf{z}_{teacher}/\tau), softmax(\mathbf{z}_{student}/\tau))] \tag{61}$$

The transfer benefit satisfies:

$$\Delta_{transfer} \leq C_{KL} \cdot \mathbb{E}[KL(\mathcal{P}_{teacher}^{(target)}, \mathcal{P}_{student}^{(target)})] + \mathcal{O}(\tau^{-1}) \tag{62}$$

Step 5: Attention Mechanism Consistency

The multi-head attention ensures consistent feature importance across domains:

$$\|Attention_{source}(\mathbf{Q}, \mathbf{K}, \mathbf{V}) - Attention_{target}(\mathbf{Q}, \mathbf{K}, \mathbf{V})\|_F \leq \rho \cdot \mathcal{W}_2(\mathcal{P}_{source}, \mathcal{P}_{target}) \tag{63}$$

for some Lipschitz constant ρ.

Step 6: Transfer Learning Generalization

Using Rademacher complexity theory for transfer learning:

$$\mathbb{E}_{target}[error] \leq \mathbb{E}_{target}[empirical\ error] + 2\mathfrak{R}_{n_{target}}(\mathcal{F}) + \Delta_{complexity} \tag{64}$$

where $\mathfrak{R}_{n_{target}}(\mathcal{F})$ is the Rademacher complexity on target domain.

Step 7: Multi-Task Learning Benefit

The shared representation learning provides:

$$\mathbb{E}_{task}[\varepsilon_{task}] \leq \min_{task} \varepsilon_{task} + \frac{C_{mtl}}{\sqrt{T}} \sum_{t=1}^{T} Similarity(task, t) \tag{65}$$

where T is the number of source tasks.

Step 8: Climate Adaptation Consistency

Climate-aware features ensure consistent performance across weather conditions:

$$|\mathbb{E}[\varepsilon|\mathbf{W} = \mathbf{w}_1] - \mathbb{E}[\varepsilon|\mathbf{W} = \mathbf{w}_2]| \leq L_{climate}\|\mathbf{w}_1 - \mathbf{w}_2\|_2 \tag{66}$$

for Lipschitz constant $L_{climate}$.

Step 9: Domain-Specific Error Decomposition

The domain-specific error ε_{domain} decomposes as:

$$\varepsilon_{domain} = \underbrace{\mathbb{E}[|\mathbb{E}[E|X, D = target] - \mathbb{E}[E|X, D = source]|^2]}_{concept\ shift} + \underbrace{Cov(E, D|X)}_{domain\ dependence} \tag{67}$$

Step 10: Final Adaptation Bound

Combining all components:

$$\mathbb{E}_{target}[|\hat{E}_t^{final} - E_t|^2] \tag{68}$$

$$\leq \min_d \mathbb{E}_{source,d}[|\hat{E}_t^{stage1} - E_t|^2] + \underbrace{C_{KL} \cdot KL_{distill} + \rho \cdot \mathcal{W}_2(\mathcal{P}_s, \mathcal{P}_t)}_{\Delta_{transfer}} \tag{69}$$

$$+ \underbrace{L_{climate}\mathbb{E}[\|\mathbf{W}_{target} - \mathbf{W}_{source}\|_2] + Cov(E, D|X)}_{\varepsilon_{domain}} \tag{70}$$

This establishes the adaptation effectiveness bound.

Corollary 2 (Transfer Learning with Limited Target Data) *For practical deployment scenarios with limited target domain data, the framework maintains robust prediction accuracy through effective knowledge transfer, with performance characteristics given by:*

$$\mathbb{E}_{target}\left[|\hat{E}_t^{final} - E_t|^2\right] \leq \mathbb{E}_{target}\left[|\hat{E}_t^{stage1} - E_t|^2\right] - \Delta_{improvement} + O\left(\frac{1}{\sqrt{N_{target}}}\right) \tag{71}$$

where $\Delta_{improvement} > 0$ quantifies the empirically observed enhancement from the second stage processing, and the $O(1/\sqrt{N_{target}})$ term reflects the standard statistical learning behavior with respect to target domain sample size, consistent with practical machine learning deployment patterns.

Proof 4 *We establish the limited data transfer performance through sample complexity analysis, statistical learning theory, and empirical improvement quantification.*

Step 1: Sample Complexity in Transfer Setting

For the target domain with N_{target} samples, the empirical risk satisfies:

$$\left|\mathbb{E}_{target}[\ell(\hat{f},E)] - \frac{1}{N_{target}}\sum_{i=1}^{N_{target}} \ell(\hat{f},E_i)\right| \leq 2\mathfrak{R}_{N_{target}}(\mathscr{F}) + \sqrt{\frac{\log(1/\delta)}{2N_{target}}} \tag{72}$$

with probability $1-\delta$.

Step 2: Rademacher Complexity for Transfer Learning

The Rademacher complexity in transfer setting is bounded by:

$$\mathfrak{R}_{N_{target}}(\mathscr{F}_{transfer}) \leq \mathfrak{R}_{N_{target}}(\mathscr{F}_{base}) + \frac{C_{transfer}}{\sqrt{N_{target}}}\mathscr{C}(\mathscr{F}_{source}, \mathscr{F}_{target}) \tag{73}$$

where $\mathscr{C}(\mathscr{F}_{source}, \mathscr{F}_{target})$ measures the complexity of the transfer mapping.

Step 3: Improvement from Second Stage Processing

The LSTM-attention-transfer enhancement provides:

$$\Delta_{improvement} = \mathbb{E}[|\hat{E}^{stage1} - E|^2] - \mathbb{E}[|\hat{E}^{final} - E|^2] \tag{74}$$

This improvement comes from enhanced feature extraction and attention weighting:

$$\Delta_{improvement} \geq C_{attention} \cdot \mathbb{E}[Var(Attention(\mathbf{h}_t))] + C_{transfer} \cdot Mutual\_Info(\mathscr{P}_{source}, \mathscr{P}_{target}) \tag{75}$$

Step 4: Information-Theoretic Transfer Benefit

The mutual information between source and target domains quantifies transfer potential:

$$I(\mathbf{X}_{source}; \mathbf{X}_{target}) = \mathbb{E}_{p(\mathbf{x}_s,\mathbf{x}_t)}\left[\log\frac{p(\mathbf{x}_s,\mathbf{x}_t)}{p(\mathbf{x}_s)p(\mathbf{x}_t)}\right] \tag{76}$$

Higher mutual information implies greater transfer benefit.

Step 5: Attention Mechanism Improvement Bound

The attention mechanism provides improvement through better feature weighting:

$$\mathbb{E}[|\hat{E}^{attention} - E|^2] \leq \mathbb{E}[|\hat{E}^{uniform} - E|^2] - C_{att} \cdot Var(\alpha_t) + \mathscr{O}(d^{-1}) \tag{77}$$

where α_t are the attention weights and d is the feature dimension.

Step 6: Transfer Learning Regularization Effect

Knowledge transfer acts as implicit regularization:

$$\mathscr{L}_{target}^{transfer} = \mathscr{L}_{target}^{base} + \lambda \sum_l \|\theta_l^{target} - \theta_l^{source}\|^2 \tag{78}$$

This regularization reduces overfitting with limited target data.

Step 7: Meta-Learning Perspective

From a meta-learning viewpoint, the second stage adaptation follows:

$$\theta_{adapted} = \theta_{pretrained} - \alpha \nabla_\theta \mathscr{L}_{target}(\theta_{pretrained}) \tag{79}$$

The adaptation error satisfies:

$$\mathbb{E}[|\hat{E}(\theta_{adapted}) - E|^2] \leq \mathbb{E}[|\hat{E}(\theta_{pretrained}) - E|^2] - \alpha \mathbb{E}[\|\nabla \mathscr{L}_{target}\|^2] + \frac{\alpha^2 L}{2} \mathbb{E}[\|\nabla \mathscr{L}_{target}\|^2] \tag{80}$$

Step 8: Statistical Learning Rate
For the limited target data scenario, the learning rate follows:

$$\mathbb{E}[excess\ risk] = \mathscr{O}\left(\sqrt{\frac{d \log(N_{target})}{N_{target}}}\right) \tag{81}$$

for d-dimensional models, which is the standard rate.
Step 9: Domain Adaptation with Few Samples
The domain adaptation error with limited target data satisfies:

$$\varepsilon_{DA} \leq \varepsilon_{source} + \frac{C_{DA}}{\sqrt{N_{target}}} + \mathscr{O}(d_{\mathscr{H}\Delta\mathscr{H}}(\mathscr{P}_s, \mathscr{P}_t)) \tag{82}$$

Step 10: Final Limited Data Transfer Bound
Combining all improvements and sample complexity:

$$\mathbb{E}_{target}[|\hat{E}_t^{final} - E_t|^2] \tag{83}$$

$$\leq \mathbb{E}_{target}[|\hat{E}_t^{stage1} - E_t|^2] - \underbrace{C_{att} Var(\alpha_t) + C_{transfer} \cdot I(\mathbf{X}_s; \mathbf{X}_t)}_{\Delta_{improvement}} \tag{84}$$

$$+ \underbrace{2\mathfrak{R}_{N_{target}}(\mathscr{F}) + \sqrt{\frac{\log(1/\delta)}{2N_{target}}}}_{O(1/\sqrt{N_{target}})} \tag{85}$$

This establishes the corollary with the specified improvement and sample complexity terms.
